# Impact of endometriosis on women’s lives: a qualitative study

**DOI:** 10.1186/1472-6874-14-123

**Published:** 2014-10-04

**Authors:** Maryam Moradi, Melissa Parker, Anne Sneddon, Violeta Lopez, David Ellwood

**Affiliations:** PhD candidate at the Australian National University School of Medicine, Canberra, Australia; Endometriosis Clinic, Canberra Hospital, Canberra, Australia; School of Medicine, and Gold Coast University Hospital, Griffith University, Kragujevac, Queensland Australia; Yong Loo Lin School of Medicine, National University of Singapore, Singapore, Singapore

**Keywords:** Endometriosis, Pain, Qualitative research, Quality of life, Women’s health

## Abstract

**Background:**

This study aimed to explore women’s experiences of the impact of endometriosis and whether there are differences across three age groups.

**Methods:**

A qualitative descriptive design was conducted using semi-structured focus group discussions with 35 Australian women with endometriosis, in three age groups. All tape-recorded discussions were transcribed verbatim and read line by line to extract meaningful codes and categories using NVivo 9 software through a thematic analysis approach. Categories were then clustered into meaningful themes.

**Results:**

Participants’ ages ranged from 17 to 53 years and had a history of 2 to 40 years living with endometriosis, with an average delay time to diagnosis of 8.1 years. Two main themes emerged: (1) experiences of living with endometriosis, and (2) impact of endometriosis on women’s lives, with 14 discrete categories. The results showed similarities and differences of the impact between the three age groups. The most highlighted impacts were on marital/sexual relationships, social life, and on physical and psychological aspects in all three age groups, but with different orders of priority. Education was the second most highlighted for the 16–24 years, life opportunities and employment for the 25–34 years; and financial impact for those 35 years and above.

**Conclusions:**

Our findings show that endometriosis impacts negatively on different aspects of women’s lives. A better understanding of these findings could help to decrease the negative impact of endometriosis by guiding service delivery and future research to meet more effectively the needs of women and teenagers with this condition.

## Background

Endometriosis is a chronic disease, which is under-diagnosed, under-reported, and under-researched [1]. It is defined as the presence of endometrial tissue outside the uterus and is found in women of all ethnic and social groups [2]. The prevalence has been reported around 10% of the general female population [2, 3] and 20-90% in women with pelvic pain or infertility [2]. However, the aetiology and pathogenesis is not known with certainty [4]. Endometriosis is often labeled ‘the missed disease’ [5] and the average time between onset of pain and diagnosis is nearly 8 years in the United Kingdom, and 12 years in the United States of America [6].

There is no cure for endometriosis and no guarantee that it will not return [7]. The economic burden is high and similar to other chronic diseases such as diabetes, Crohn’s disease and rheumatoid arthritis [8]. A survey across 10 countries estimated that the average annual cost of endometriosis was €9579 per woman consisting of €3113 for health care costs and €6298 for productivity losses [8]. Many patients’ quality of life is affected by pain, the emotional impact of sub-fertility, anger about disease recurrence, and uncertainty about the future regarding repeated operations or long-term medical therapy [2]. A recent study conducted by Nnoaham et al. [9] identified impaired health related quality of life and work productivity across countries and ethnicities, yet women continue to experience delay in diagnosis. From the patients’ view, endometriosis can be a nightmare of misinformation, myths, taboos, lack of diagnosis, and problematic hit-and-miss treatments overlaid by a painful, chronic, stubborn disease [10]. The impact includes fertility, sexuality, ability to work, play, and personal relationships [10].

Qualitative research has been undertaken to explore women’s experiences of living with endometriosis [11–14] and to explore its impact on quality of life [15], or on chronic pain [16], diagnosis [17], experiences in the primary care setting [18], social and working lives [19], dyspareunia and relationships [20]. A systematic review of evidence revealed that little qualitative research has been conducted to explore the reality of living with the condition, and many of these studies lacked rigour [21]. A recent narrative review study on social and psychological impact of endometriosis mentioned that virtually nothing is known about how demographic characteristics like age impacts on the experience of endometriosis [22]. In addition, there are few qualitative findings on teenagers. Therefore, we conducted a study to explore women’s experiences of endometriosis and its impact, involving three different age groups recruited either from both a hospital clinic and the community.

## Methods

A qualitative descriptive design was used for this study. This study was granted approval from the ACT Human Research Ethics Committee (ETH.11.10.395) and the ANU Human Research Ethics Committee (Protocol: 2011/049). This study adheres to the RATS guidelines (http://www.biomedcentral.com/authors/rats).

### Participants

A sample of 35 women was purposefully recruited including 23 women from a dedicated Endometriosis Centre at one public teaching hospital in Canberra and 12 women from the community (who had not attended the Centre). The women from the community centre were recruited to avoid a potential bias for recruiting more severe cases from a dedicated Endometriosis Centre and to increase generalizability of our findings. Women were included with a confirmed diagnosis of endometriosis (via laparoscopy) for at least a year, who were able to understand and speak English, and had no other chronic disease. Through a dedicated Endometriosis Centre eligible women were contacted by telephone and invited to participate. An information sheet explaining the purpose and nature of the study was sent by email to women who were interested in taking part. To recruit from the community, eligible women were identified through the general practice software that records the primary diagnosis. A doctor within the practice extracted a list of names with the diagnosis of endometriosis throughout 2011, and then posted an invitation letter to those who met the eligibility requirements. Recruitment was also attempted through an ‘Endometriosis Information Night’ in May 2012 conducted in Canberra. The study was introduced at that event and an invitation letter with an information sheet were provided, and interested women were invited to provide contact details, or respond later through a stamped addressed envelope. Ten focus group discussions with 3 to 4 participants were conducted. The focus groups consisted of three distinct age groups: Group 1(16–24 years); Group 2 (25-34years); and Group 3 (35 years and above). It is assumed that a more homogenous group provides the participants with greater freedom to express thoughts, feelings, and behaviors candidly [23].

A pilot with 4 women was conducted to test the appropriateness of the interview questions and length of time needed. The pilot focus group lasted for 2.5 hours and it was determined that four to five women for each focus group would be manageable to give everyone the opportunity to share their experiences [24]. Small focus groups are more comfortable for participants and preferable when the participants have a great deal to share about the topic or have had intense or lengthy experiences with the topic of discussion [25].

### Procedure

Semi-structured, in-depth focus group discussions were used as the method of data collection. Polit and Beck [26] suggest semi –structured interviews are used when researchers have a list of topics or broad questions that must be addressed in an interview. Before the session, written informed consent was obtained and participants were asked to complete a demographic and clinical questionnaire. To ensure anonymity numbers were allocated to the participants and no names were used during the discussions. Interview questions were developed based on a literature review that explored the experience of women of endometriosis, and assessed the impact of symptoms. An interview guide was constructed with two main research questions of ‘How are women’s experiences of living with endometriosis?’ and ‘How does endometriosis affect women’s lives?’, but the process remained flexible to follow up interesting discussions that emerged. All discussions were tape-recorded, and facilitated by two experienced health professionals with practical knowledge about endometriosis and interviewing skills. The facilitators function was to encourage participants to talk freely [26]. Discussions took 2 to 2.5 hours with an average recording length of 122 minutes. Recruitment of participants continued until data saturation was reached when new participants no longer produced new insights [27]. This study took 27 months to complete from September 2010 to December 2012.

### Data analysis

All recordings of focus group discussions were transcribed by an accredited, transcribing company. Data analysis began after the first focus group discussion using NVivo 9 software. Six phases of thematic analysis were used as described by Braun and Clarke [28] including: (1) familiarizing yourself with your data, (2) generating initial codes, (3) searching for themes, (4) reviewing themes, (5) defining and naming themes and (6) producing the report. The lead researcher began the analysis by listening to the voice recording and reading and re-reading the transcriptions. Data were coded according to related points in the transcription followed by categorizing the codes to themes. The themes were checked against the codes extracted before defining the themes supported by extracts from the transcriptions. The analytical process was verified by the research team by reviewing all field notes, coding data, and themes. Analysis was conducted at group and individual levels with consideration given to women’s demographic information.

Rigour refers to the quality of qualitative enquiry and is used as a way of evaluating qualitative research [29]. Seven participants from different focus groups were asked to check a transcription of their responses and confirmed its accuracy. Production of counts of phenomena, searching for deviant cases, comparison within and across age groups, and team working was accomplished using the NVivo software [30]. Systematic field notes improve reliability in qualitative research [27]. The lead researcher took field notes during and immediately after each discussion and during the analytic process to keep a record of the data coding steps. In addition, another team investigator participated in all focus group discussions and shared her field notes, which concurred with the lead researcher’s notes.

## Results

### Demographic and clinical findings

The mean age of the participants was 31.1 ± 10.4 years (range 17–53). Most (30 out of 35) were Australian-born, except one from New Zealand, one from Asia, two from Europe and one from Africa. Most were married or had partners and had a history of 2 to 40 years living with endometriosis. The women reported that their first endometriosis-related symptoms were experienced at a mean age of 17.4 ± 6.8 years (range: 11–41), and diagnosis was made at 25.6 ± 7.9 years (range: 15–42), with an average of 8.1 ± 6 years (range: 3 months - 24 yrs) delay in diagnosis of endometriosis. Almost half (17 out of 35) of the participants reported that endometriosis interferes ‘a lot’ with their life and 54.3% (19 out of 35) had ‘moderate’ satisfaction with their treatment (Table 1).

**Table 1:** **Characteristics of participants (n = 35)**

Characteristics		n (%)
Age (years)		31.1 ± 10.4^a^, range: 17- 53
	16-24 years	13(37.1)
	25-34 years	11(31.4)
	35 and above years	11(31.4)
Educational level	Secondary	19(54.3)
	Undergraduate	10(28.6)
	Postgraduate	6(17.1)
Employment	Full time	26(74.3)
	Part time	6(17.1)
	Unemployed	3(8.6)
Marital status	Married/partner	25(71.4)
	Single	9(25.7)
	Divorced	1(2.9)
Obstetric history	Nulliparous	22(62.9)
	Live birth	9(25.7)
	Termination	4(11.4)
	Miscarriage	4(11.4)
Age at onset of symptoms (years)		17.4 ± 6.8^a^, range: 11-41
Age at diagnosis (years)		25.6 ± 7.9^a^, range: 15-42
Delay in diagnosis (years)		8.1 ± 6^a^, range: 3 months-24 yrs
Common symptoms	Period pain	34(97.1)
	Heavy bleeding	27(77.1)
	Dyspareunia	25(71.4)
	Bowel pain	25(71.4)
	Irregular bleeding	24(68.6)
	Bladder pain	21(60)
	Infertility^b^	10(58.8)
	Non menstrual pain (lower abdomen, back or leg pain)	8(22.9)
Endometriosis interference with their life	A little	2(5.7)
	Moderate	6(17.1)
	A lot	17(48.6)
	Very Much	10(28.6)
Treatment satisfaction	Not at all	2(5.7)
	A little	6(17.1)
	Moderate	19(54.3)
	A lot	6(17.1)
	Very much	2(5.7)

^a^Mean ± SD.

^b^Infertility was not applicable to 18 participants, so it was calculated out of 17 participants and 10 out of 17 were infertile.

### Qualitative findings

Experiences of living with endometriosis were similar between the hospital-based and community-based groups. Two main themes and 14 categories emerged from the data as shown in Table 2. The two main themes were: (1) experiences of living with endometriosis, and (2) impact of endometriosis on women’s lives. The results also showed the similarities and differences of the impact of endometriosis between the three age groups (Table 3). The most highlighted impacts for all the three groups were on marital/sexual relationship, social life, physical and psychological impact but with different orders of priority. However, some differences in the next most highlighted impact were noted, with education being most important for the 16–24 years, life opportunities and employment for the 25–34 years; and financial impact for the 35 and above years old women.

**Table 2:** **Themes and categories that emerged from the data**

Themes	Categories
Experiences of living with endometriosis	1. Symptoms related to endometriosis
	2. Delayed diagnosis
	3. Treatment of endometriosis
	4. Experience with health care providers
	5. Lack of information
Impact of endometriosis on women’s lives	1. Physical impact
	2. Psychological impact
	3. Marital/sexual relationship impact
	4. Social life impact
	5. Impact on education
	6. Impact on employment
	7. Financial impact
	8. Impact on life opportunities
	9. Impact on lifestyle

**Table 3:** **Most highlighted impact of endometriosis for the different age groups**

Age group	Group1 (16–24 years)	Group2 (25–34 years)	Group3 (35 and above years)
**Similarities**	1- Social life	1- Marital/Sexual relationship	1- Physical impact
	2- Marital/Sexual relationship	2- Psychological impact	2- Marital/Sexual relationship
	3- Physical impact	3- Physical impact	3- Psychological impact
	4- Psychological impact	4- Social life	4- Social life
**Differences**	• Education	• Life opportunities	• Financial impact
		• Employment	

### Theme 1: experiences of living with endometriosis

#### Symptoms related to endometriosis

The most commonly experienced symptoms were pain, dypareunia, heavy/irregular bleeding and infertility. All women had suffered severe and progressive pain during menstrual and non-menstrual phases in different areas such as the lower abdomen, bowel, bladder, lower back and legs that significantly affected their lives. Other symptoms were fatigue, tiredness, bloating, bladder urgency, bowel symptoms (diarrhoea), bladder symptoms and sleep disturbances due to pain.

“*I think I was about 14 years old when I had the symptoms. Yeah, lots and lots of pain and I couldn’t move. There was always constant pain. I didn’t have a day without pain. I used to have days off because of it. I just sat there and could not move and I cried”.* (P21, Group 1)

The women described the pain as ‘sharp’, ‘stabbing’, ‘horrendous’, ‘tearing’, ‘debilitating’ and ‘breath-catching’. Severe pain was accompanied by vomiting and nausea and was made worse by moving or going to the toilet. The frequency of pain differed between the women with some reporting pain every day, some lasting for three weeks out of each menstrual cycle, and another for one year.

*“I remember getting the first lot of symptoms like someone had heated a knife and was ripping it up through my stomach. That’s how it felt. I didn’t know how else to explain it”.* (P15, Group 2)

Most of the women complained of dyspareunia during and/or after sex although this was a more important issue for Group 2.

*“I started to worry when my ex-partner and I got together and the pain during and after sex just got that bad that I would just lay in a fetal position for hours afterwards. It got progressively worse to the point where I would actually be crying during and after sex”.* (P5, Group 2)

Heavy and/or irregular bleeding was another symptom experienced but in some women, it was a side effect of endometriosis treatment. Bleeding when exercising and after sex were experienced by only a few women. Women and their partners were particularly worried when bleeding occurred after sex.

*“It’s the bleeding, the constant bleeding that is the most frustrating part because I have to be stocked up with pads, have a spare of everything because I just don’t know when it’s going to happen”.* (P3, Group 2)

### Delayed diagnosis

Delay between the start of symptoms until diagnosis ranged from three months to 24 years with an average of 8.1 years. Usually endometriosis was diagnosed after years of delay and with women already experiencing severe symptoms. The average delay to diagnosis was 4.9 years, 8.2 years, and 11.9 years for Groups 1, 2, and 3 respectively. Most women reported visiting a variety of health professionals and undergoing a whole range of tests, but without being diagnosed with endometriosis. In some cases, they were misdiagnosed and mistreated for appendicitis, ovarian cyst, ectopic pregnancy, pelvic inflammatory disease, and ovarian cancer.

*“From the time I was 13, I went through a number of different doctors to try and find the problem, most just told me that some people have heavier periods than others and more pain and don’t cope well with the pain, and that it was normal. He basically called me a ‘sook’ [sic] without putting it in those words. So I did go through a lot of doctors and frustrations. I even got banned from some doctors’ surgeries for getting angry at them for not listening. There was a lot of tests done in the meantime before I got diagnosed… scary thoughts like ectopic pregnancies…. I didn’t get diagnosed until I got referred through another specialist and had my first surgery”.* (P22, Group 1)

The reasons for the delayed diagnosis related to patient, family, friends and colleagues, physician, and the health system. The women reported that they normalized their severe period pain or other symptoms and did not take their symptoms seriously. They were thinking things like “I am not fortunate that I have painful periods”, “it is a part of womanhood”, and did not like to share or talk with others about their symptoms because of shame. Their family and friends also told them that pain and bleeding were normal, and their doctors misdiagnosed or mistreated them because they normalized symptoms and did not believe them, or they lacked knowledge.

*“I know I suffered with the pain and it was bad. But my GP [family doctor] I’d been telling him since I was 13 years old something was wrong, and he was male and he just didn’t want to follow it up…he sort of just brushed it off – you’re OK. Put me on a tablet called Ponstan and I was on that. He just kept telling me to take that you’ll be fine, you’ll be fine and everyone pretty much thought I was just being a sook [sic] until I was diagnosed at 21”.* (P18, Group 1)

Difficulty accessing a gynaecologist and long surgery waiting lists were reported by participants, and these also contributed to a delay in diagnosis. Some women reported that they resorted to self-diagnosis because of a known family history, information from the Internet or informed friends, and interaction with other patients with endometriosis.

### Treatment of endometriosis

The women reported receiving different medical and surgical treatments. In addition to current medical and surgical treatments, complementary or alternative therapy and lifestyle changes such as exercise, diet and good sleep were used to manage their endometriosis.

*“I’ve tried the treatments, surgeries, steroids …and the pill but I’m allergic to the pill now. I’ve just had 13 changes to the pill in one year and 13 pregnancy tests, all of which were negative. So the year before they discovered I was allergic to the pill. And also [tried] alternative medicines, naturopathies which [I found] the combined effect, was the most effective”.* (P7, Group 3)

Some women reported that the doctor recommended them to have a baby at an early age. Surgery for the majority of women had a significant role in relieving pain and other symptoms but they experienced recurrence of disease causing negative psychological and emotional impacts. Two of the women in Group 3 underwent hysterectomy at age 37 and 38. Another two women from Groups 1 and 2 tried to convince their doctors to perform a hysterectomy to get rid of their symptoms. For some, treatments were ineffective and about half had experienced side effects like breakthrough bleeding, depression and mood swings, or feeling sleepy at work because of the analgesic effects. Some women also reported feeling frustrated with their constant use of pain killers, and were fearful of becoming tolerant to pain killers and the need to increase the dose.

### Experience with health care providers

Women reported both negative and positive experiences with health professionals, but negative experiences were mostly highlighted. Most of the negative experiences were related to health professionals who did not want to listen to their concerns, had no time to answer their questions, and told them that the symptoms they experienced were ‘normal’ and ‘not serious’. They also reported difficulty in accessing a specialist who understood endometriosis. The majority of the teenagers reported that the physicians did not take their symptoms seriously, did not believe them, and thought that they were only making up stories just to get attention. Some reported that doctors did not believe that endometriosis was a problem that affected young women.

*“I found it very hard to access my specialists. I was in so much pain that I called my specialist and I just wanted some guidance as to what I could do. The answer was, “Have some more Panadol”. My experience in Sydney wasn’t that great either. I went to a specialist in Sydney. I had a lot of questions and thought that because he is a specialist having to deal with hundreds and hundreds of people that he can give me the answers to my questions but he did not have time to talk”.* (P1, Group 3)

On a positive note, some women reported that their doctors were really sympathetic, understanding, and had time to talk. The team at the dedicated Endometriosis Centre was reported to be caring, supportive and accessible.

### Lack of information

Many women reported that they had not heard about endometriosis prior to diagnosis. The lack of information was apparent before and after their diagnosis.

*“Yeah, I should be aware but it was kept as a secret, I didn’t know that this exists. I’m a quite a sort of [a] nosey person in terms of health and what you can catch and what you can’t. In school, we’ve never heard of endometriosis… even when I did biology and science. I just never heard anything especially when teenagers are so sexually active and you just want them to know what was going on”.* (P19, Group 1)

Most women were dissatisfied for not being given enough information at the time of diagnosis. Some women reported that the doctors’ lack of information about diagnosis of endometriosis may have caused delayed diagnosis.

*“My doctor did not have a great deal of knowledge in that area. With two of my doctors, I had to explain to them what endometriosis is and how to treat it. So you can’t exactly go to them and ask for help with my treatment”.* (P22, Group 1)

### Theme 2: impact of endometriosis on women’s lives

Most women reported that endometriosis had significant impacts as they lived through it every day of their lives. Women used the following expressions to describe the impact:

*“Living with it, it does affect me nearly every day I feel it”.* (P18, Group 1)

*“It has an impact in every aspect of your life”.* (P9, Group 2)

*“It has a big impact”.* (P6, Group 3)

“O*verall endometriosis has made me live a very solitary life”.*(P26, Group 2)

### Physical impact

The physical impact was associated with symptoms, treatment side-effects and changes in physical appearance. Pain in particular was reported to limit their normal daily physical activity like, walking and exercise.

*“Prior to having endometriosis, I used to run every day and was very active. For the last six months while I waited for surgery to remove the cyst and endometriosis, my level of exercise was severely reduced. I could only walk about once or twice a week and it was very painful. I gained weight and felt dissatisfied with my body. It also impacted on my self-confidence. I began wearing more baggy clothes, also watched more TV and had general feelings of being lethargic and spaced out”.* (P1, Group 3)

Women who had small children mentioned that they were not able to care for them as they would like. Some women were not satisfied with their body appearance because they had gained weight, or had scars from surgery, or were pale because of heavy bleeding and anemia. Fatigue and limited energy were also among reported physical impacts of endometriosis. Although infertility was primarily a physical impact of endometriosis, it had a negative impact on the psychological health, relationship, and financial status of the women.

### Psychological impact

Most women experienced feeling upset, angry, depressed, uncertain, weak, powerless, helpless, hopeless, defeated, disappointed, frustrated, exhausted, and felt like a burden to others.

*“So the negative effects can be quite huge. And I even feel depressed with myself. I feel angry with myself and I go I don’t want to deal with it anymore. So I’m like I’m not on the diet any more. I still smoke. And I just, I mean I’ll have it for the rest of my life anyway so why do these things to, it’s not going to prevent it, it’s not going to make it go away, why can’t I eat what want?”* (P17, Group 2)

Some women reported that endometriosis had interfered with their identity in the following ways: not being able to have sex and feeling like “I am not a woman”; being infertile; not being a good mother or the mother that they wanted to be; not able to do simple daily activities; not feeling happy; and feeling like “it is not me”.

*“The psychological damage that [this] has caused me is immense. It’s horrible… I feel like I’m not even a woman. What’s the point of even being a woman? I feel like I’m denied some part of being human. Like for example, if someone couldn’t go to the toilet, it’s a basic human act and function and I’ve been denied that”.* (P11, Group 2)

A negative effect on self-esteem, self-confidence and lack of control of their life (powerlessness) were other reported psychological impacts. Some women reported negative impacts on self-confidence because of, not being able to have a child or have more children, not able to have normal sex, being unsatisfied about body appearance because of weight gain or lots of scars. At the time of diagnosis, about half experienced a kind of relief because they found the reason for their problems, or found that it was not in their head and they were not mad, or knew the name of the condition causing their symptoms, and hoped that they could try to solve it and that they did not have something severe or untreatable like cancer. However, some were upset, overwhelmed or worried when they found that the problem had no cure or there was a risk of infertility. Women reported that obtaining their diagnosis took ‘too long’ and it was ‘too late’, and some were upset, angry, frustrated and annoyed because it took so long to be diagnosed and they were not listened to or believed or understood by doctors, family, friends and colleagues and at being labeled inappropriately (e.g. suffering from sexually transmitted infections).

*“I was in a way relieved because you have the answers like there was something wrong and that’s why you are experiencing what you are, plus it wasn’t sort of like an ectopic pregnancy and other things that I’d been really scared about up until being diagnosed. At the same time a bit overwhelming that it was something that was going to stay there, that it wasn’t just something that could be treated with antibiotics. Plus the risk it has to your fertility and stuff like that. That kind of overwhelmed me quite a lot to start with”.* (P22, Group 1)

Recurrence of disease was stated as the biggest fear, and women reported unpredictable recurrence of symptoms and disease prognosis leading to feelings of uncertainty.

*“It’s just unpredictable. It may recur at any time in your life. I just feel tired and shattered because you just don’t know how to deal with it and you don’t know when it’s coming back. I want to plan something like a holiday but I cannot plan it because you don’t know what’s going to happen. You just feel like you can’t really arrange your life, you are arranging your life around the disease. It controls you even if you don’t want it to”.* (P16, Group 2)

Women also discussed their concerns related to fertility (future fertility, having first child or more children), recurrence of disease, disease prognosis, worsening symptoms, interference with education, employment, sexual/marital life and motherhood responsibilities, financial concern because of losing their job and high treatment costs (like IVF), finding a new partner (because of dyspareunia or bleeding with sex), passing endometriosis on to a future daughter and getting cancer. A few women were concerned about having attacks of pain in public, or at an airport causing them to miss a flight. A number of participants were worried about leaking because of heavy bleeding or unpredictable spotting, and they were also worried about wearing white pants, sitting on white couches or sleeping on white mattresses. One participant said that she was worried about carrying lots of drugs or painkillers in her bag because of how others may judge her. Two mothers who had shared custody with their ex-husbands were worried about caring for their child while having severe pain or surgery scheduled, and were worried about losing their child custody eligibility because of being too sick or in too much pain and relying on lots of pain killers. Group 1 reported the highest number of concerns compared to the other two groups. Most teenagers were worried about their future fertility. The teenagers reported that they were not able to talk on this issue with their families or partner and they thought support groups would be very helpful. One third of women among Groups 2 and 3 reported regrets because of living with endometriosis, and delayed diagnosis was found to be the main reason for this. Other reasons for regrets were not being proactive in the diagnostic process, not being able to have an intimate relationship and satisfactory sex life (especially Group 2), not being able to have children, not having good disease management because of lack of information, and regrets about passing endometriosis on to their daughters.

### Marital/sexual relationship impact

Most women who were married or had partners reported negative impacts on their sexual relationships. This was usually because of pain during or after sex, and lesser frequency because of bleeding during or after sex.

*“I have had dyspareunia my entire life. I have never, ever had sex that wasn’t painful. I’m nearly 30 years old. I want to have sex. I mean it affects my relationships; I’ve never had one that’s lasted longer than six months because guys - you go well - especially when I didn’t know what it was, no one wants to go near that”.* (P11, Group 2)

Decrease in frequency of intercourse, avoiding having sex for fear of painful intercourse or bleeding, and failure to have orgasm caused frustration and put a strain on their relationships and some experienced relationship breakdown. Infertility or probable infertility was also reported to impact on marital relationships, and was a concern and a threat for breaking up a relationship. About half of the women reported that they did not have a supportive and/or understanding partner. In some cases, not being understood by their partners was a reason for argument and a broken relationship. Due to the negative impact of endometriosis on marital/sexual relationship some were anxious about initiating a new relationship and a few women had chosen to stay single.

*“I have been married and the marriage broke up. One of the aspects was because I couldn’t fall pregnant plus I guess I was moody and in a lot of pain and he couldn’t put up with that. … I don’t feel it’s fair to bring another guy into my life who’s fit, young and healthy when I might have pain, I’m tired and you know all we’re going to do is cause arguments and I just don’t have the energy to fight them. So it’s just easier to be on my own”.* (P26, Group 2)

### Social life impact

Most women reported a reduction in social activity, and opted to stay home, and missed events because of severe symptoms especially pain, bleeding and fatigue. They resorted to using up their annual leave after exhausting their sick leave because of their disease. Some women also decreased their sport or leisure activities and some gave up their routine sport including water ski, horse-riding, swimming and snow skiing.

*“I don’t tend to socialize and keep to myself because of pain and bleeding. As a result, I have missed out on travel, concerts, weekends away and school/university events etc”.* (P9, Group 2)

Some women believed that pain, stress, anger and mood swings caused by endometriosis can seriously affect relationships with others. For instance, one participant described that she became ‘cranky’ when she was in pain. Two women reported that they had a fight in the workplace because of negative impacts of endometriosis on their mood, especially pain and fatigue.

*“Just because yeah, it’s stressful and you’re angry and I guess that’s the point where it can affect your relationships with people more seriously”.* (P12, Group 1)

Some women distanced themselves from any social events and gatherings with family and friends because they felt that they were different or felt jealous of others. A few women reported feelings of isolation.

*“But as far as social activities go, I have completely distanced myself from social activities. I guess in a way distancing myself limited my opportunities of interacting with eligible men. I don’t want to be around happy couples who are mostly my friends. They’re all happily married. I don’t want to be around them because it just reminds me of what I don’t have”.* (P11, Group 2)

### Impact on education

Education was the main issue for teenagers (Group 1). Two thirds of women reported an impact on education such as taking time off school (even for one whole semester), finding it hard to focus and being less productive in schoolwork. Some reported obtaining bad sports grade, having to study part time and defer university, and one teenager actually left school because of her disease.

*“I was missing a lot of time off school and they thought it’d be a good idea to have surgery. I waited about three months for the surgery and then it got to the point where it got really, really bad where I had three weeks straight off from school”.* (P17, Group 2)

Some participants, however, were determined to stay in school despite symptoms and so they were taking more medication to overcome symptoms. Some women had to schedule their surgery during school holidays and some opted to study by correspondence.

### Impact on employment

A negative effect on employment was the main issue for Groups 2 and 3. Having endometriosis led them to take time off work, choose part-time work, made them less productive, whilst some had to give up their favorite job, or lost the chance of promotion.

*“I left my part time job because I was not able to work due to severe symptoms and undergoing two surgeries… Having two surgeries within a year it’s kind of hard to find a job if you think that that’s going to be ongoing, not many people are going to employ you to have time off”.* (P22, Group 1)

In addition, for some there was an impact on employment options and it limited what they could do in the work place. Some were forced to go to work even with severe symptoms because they found it difficult to take time off. Participants who did not have an understanding employer had more negative experiences and faced threats of losing their job.

*“I guess I’m lucky in my employer… working for the public service quite understands. Prior to that though I was in private enterprise and that wasn’t quite as understanding and used to talk to me and I would potentially lose my job if I had any more sick days”.* (P9, Group 2)

### Financial impact

Financial impacts were mainly expressed by Group 3, firstly from the cost of treatment such as medications (especially some that were not covered by the Pharmaceutical Benefits Scheme), surgery, infertility treatment, complementary therapy (e.g. naturopathy, psychology or sex therapist), and sanitary pads. The second impact related to loss of, or decrease in income due to working part time, no paid sick leave and taking time off, or losing the chance of working during school holidays because they had to schedule their surgery at that time.

*“Financially, it impacted [on] me quite a lot because when I was put on the pill the ones that were covered by the pharmaceutical benefits…The only one I found worked for me wasn’t covered and it was quite expensive”.* (P22, Group 1)

*“Financial [impact]; massive, because you’re taking so much time off work. … There’s no way you’re getting out of bed that day and just not getting up and coming, not being able to pay those bills, it does put a massive stress on you”.* (P33, Group1)

Among the women who did not mention a negative financial impact, most were from Group 1 who were being supported by their family and did not have financial responsibilities, and a few of them were working full time and were paid for sick leave.

### Impact on life opportunities

Participants reported negative impact on their life opportunities, which in two thirds of cases were related to employment, such as giving up or not being able to be employed in their favorite job, losing the chance for promotion, choosing a less stressful job and even getting fired. In one third of cases the impact on life opportunities were reported as broken relationships or engagement. Two women gave up the opportunity to compete at elite sport, and two were not able to continue their education (leaving school and postponing university). Group 2 were mostly affected by life opportunities.

*“I’ve been an elite athlete [Sport name] on [Name] team since I was 14 …. Pain, tiredness and fatigue due to endometriosis had big impact on my sporting life…. Obviously it’s a big impact on that particularly during competition season so I would be on the start line. I’ve been on the start line in a competition, you know silent tears running down my face and my mum rubbing my back and it’s [Name] championship or something and I have just got to start, so that’s had a huge impact on my life. Also in terms of obviously when I had surgery in [Year] that typically took a big chunk out of my competition season this year. So that was a big impact… So that’s definitely something that can disrupt that side of things”.* (P23, Group 1)

### Impact on lifestyle

Some women believed that there had not been any impact on their lifestyle, but among those who stated that endometriosis did affect their lifestyle, negative impacts were more highlighted than positive. Some women reported negative impacts on their lifestyle such as taking more analgesia, consuming more alcohol, smoking more cigarettes (tobacco) and in two cases using illicit drugs to help them cope with pain or feelings and their condition.

*“I did find myself taking at times more pain killers than I should and when that wasn’t working; I did at one stage try marijuana. I reckon I had been told that that did help with the pain and sleeping, because I had problems with sleeping because of the pain. But as far as drinking and stuff goes, no”.* (P22, Group 1)

Participants were also asked whether there has been any positive impact regard to living with endometriosis, and most of them reported that there has been not any positive impact.

*“It’s laughable to think that anything is good about this disease”.* (P 11, Group 2)

A few women, however, believed that there had been a positive impact of endometriosis such as choosing a healthy diet or doing exercise and giving up smoking.

*“It has had a positive effect in my lifestyle and habits. I used to be a smoker… and I just quit and like yeah it’s all good, I’m over it now. Like 18 months ago, it’s been really hard to just go no cold turkey, quit, whatever. But then I came here in July/August and all that sort of stuff needed to stop to treat it…. So I guess that’s sort of the positive impact, that sort of bad eating habits and things like that [Laughs], I had to change that”.* (P15, Group 2)

A number of women believed that they have learned lessons due to living with endometriosis (like became ‘determined’ and ‘stronger’, ‘dealing with disease instead of fighting’ and listening to their body), and that their pain tolerance had increased, and that now they can understand and help others with the same symptoms.

Participants at focus group discussions were also asked about ‘how they view the future’, and women’s views were diverse. Most women had a positive view, but believed it would be affected by their disease, and better control and disease management was their common plan. A pain free future, to have a family and an intimate relationship, less disease recurrence, and an effective treatment were their main hopes. Besides better disease management, which was expressed by women in all age groups, an educational and occupational plan for Group 1, and efforts to have a baby for Group 2 were the main priorities. In Group 3 the plans varied according to age; some still planned to have a baby and some older ones hoped to lose their symptoms by reaching menopause.

## Discussion

Our findings show that endometriosis has negative impacts on different aspects of daily life, highlighting similarities and differences between three age groups. There is a large body of quantitative studies on the psychological/psychosocial impact of endometriosis [31–35]. However, there were contradictory results on the psychological characteristics of patients with endometriosis [35]. Quantitative studies have reported that endometriosis leads to a poor or impaired quality of life [9, 36–39]. A systematic literature review by Gao et al. [1] demonstrated that endometriosis substantially affected patients’ health-related quality of life and the most often affected domains were pain, psychological functioning, and social functioning.

Previous studies were conducted on women’s experience of endometriosis. Similar to our findings, Denny [12] concluded that the experience of endometriosis pervades all aspects of a woman’s life in the UK. In addition, Jones et al. [15] reported that the impact of endometriosis on quality of life is multidimensional and more complex than just negatively affecting psychological parameters. Common themes identified through previous qualitative studies were pain, dyspareunia, delay in diagnosis [11, 14–16], and negative effects on education, work, and social and family relationships [13, 14, 19]. Diagnostic delay and uncertainty; quality of life and every day activities; intimate relationships; planning for having children, education and work; mental health and emotional wellbeing; and medical management and self-management were key categories of impact identified by a narrative review of 23 quantitative and 16 qualitative studies [22].

In our study pain was the first and most important issue discussed by most women. This finding concurred with several previous studies in which pain was constantly identified as a theme experienced by women with endometriosis [11, 12, 14–16]. In addition, review studies confirm that pain is the most important issue related to endometriosis [40, 1, 21]. In this study pain was reported to negatively impact all domains of life including physical, psychological, social, sexual, education and employment. The dominant feature of data from another study on 18 women in New Zealand was the experience of severe and chronic pain impacting on all aspects of life [16]. Jia et al. [40] based on a review study pointed that pain was strongly related to a poor ‘health-related quality of life’. Moreover, Denny [11] reported that endometriosis was the cause of severe pain for all of the women in the study and this pain affected every aspect of their lives.

The women in our study stated that obtaining their diagnosis took too long as they were not believed by doctors, family, friends and colleagues. Some were angry, frustrated and annoyed at being labeled inappropriately (e.g. suffering from sexually transmitted infections) and misunderstood. In the literature a difficult pathway to diagnosis and treatment [41] and limited effectiveness of treatments [12] were also reported. Women’s ideas of menstrual pain as ‘normal’ were shared by some doctors, and their families, resulting in further delays before a definitive diagnosis was made [17].

Some women in our study believed that pain, stress, anger, and mood swings caused by endometriosis seriously affected their relationships. Dyspareunia was a common symptom that had a major impact on sexual/marital relationships, which led to broken relationships for some women. Dyspareunia was highlighted most in Group 2. Similarly, dyspareunia was one of the key findings among several previous qualitative studies [11, 12, 14, 20]. Dyspareunia associated with endometriosis has been announced as a research priority by the World Endometriosis Society, which called it a ‘neglected aspect’ of endometriosis [42]. It has been reported that the experience of pain limits sexual activity and lack of sexual activity results in a lowering of self-esteem and a negative effect on relationships [20]. The study finding of broken relationships with partners because the strain caused by dyspareunia and avoidance of sex has been reported in other studies [12, 20]. Women had feelings of guilt or inadequacy regarding the avoidance of sex, and partners felt rejected, jeopardized or broke up relationship [20]. Fritzer et al. [43] identified statistically significant correlations between sexual dysfunction and pain intensity during/after sexual intercourse (p < 0.01/p < 0.01), a lower number of episodes of sexual intercourse per month (p < 0.01), greater feelings of guilt towards the partner (p < 0.01) and fewer feelings of femininity (p < 0.01). A recent study on heterosexual couples revealed that a negative impact of endometriosis on intimate relationships, especially sexual relations, is apparent, including but not limited to the impact of painful intercourse [44]. In some cases, living with endometriosis has strengthened bonds, and in others has led to significant strain [44].

New areas explored by our study included the impact of endometriosis on self-confidence, lost life opportunities and regrets around living with endometriosis. Concern about infertility [15, 45], employment [15], getting cancer [45], pain [14] and endometriosis in daughters [15] has been reported in previous studies. In addition, we found new areas of concern not previously reported about finding new partners, financial concerns because of losing jobs or cost of treatments, pain attacks in public, worry about leaking (because of heavy bleeding), carrying lots of drugs or painkillers, and worry about losing their child custody eligibility among single parents because of being too sick or in too much pain and relying on lots of pain killers.

There are limited studies that focus on the experiences of teenagers with endometriosis. Plotkin [45] mentioned that the diagnosis of endometriosis impinged on all aspects of adolescents’ lives such as missing out on social functions, school, and feeling different from peers. In our study, the most highlighted impact for teenagers was social life. Teenagers were mostly concerned with future fertility and some were encouraged to have an early pregnancy by doctors that made them anxious, so that one teenager stopped attending high school and decided to have an early pregnancy. Adolescent’s concerns about adult issues, e.g. future fertility was also found in another adolescent study [45].

The impact of endometriosis is worsened by a lack of understanding of the disease [46]. Many participants in our study reported that they had not heard about endometriosis before their diagnosis, and that there was a lack of information among patients, families and friends, at school and workplace. The women suggested helpful actions to decrease the negative impact of endometriosis on women’s lives which included: increasing GPs knowledge and understanding, more information for patients and increasing awareness and understanding in society such as earlier and more information at school. Most women wished that society would give more importance to endometriosis and take it as seriously as other chronic diseases like diabetes, multiple sclerosis, and cancer. The need for more support groups or networks, and a better understanding and acceptance without criticizing or stigmatizing were their further suggestions. There appeared to be greater satisfaction when services were provided by a dedicated endometriosis service, especially with getting information.

This study could be limited due to a small sample size of only 35 women. However, data saturation had been achieved which suggests that the sample size was adequate. The second limitation could be that 23 out of 35 women were recruited from one Endometriosis Centre, and that the practice provided at this clinic could be different from the other endometriosis centers. However, the results were similar for the two groups of women, which suggests that they are generalizable.

## Conclusions

In this study we have explored the impact of endometriosis on women’s lives, highlighting the similarities and differences between different age groups of women. The women recruited from both a dedicated Endometriosis Centre and from the community, reported similar negative impacts of endometriosis on different aspects of women’s daily lives. Better understanding of the long term and wide ranging impact of endometriosis on women’s lives at different life stages could benefit policy makers, health professionals and the lay population in reducing the negative impact of endometriosis and improving women’s life experiences. These findings could help to decrease the negative impact of endometriosis by guiding service delivery and future research to better meet the needs of women and teenagers with this condition. It is recommended that future qualitative research should include partners and family members of endometriosis patients. In addition, more research is warranted to explore the impact of endometriosis on adolescents.
